# Comprehensive Survey of SNPs in the Affymetrix Exon Array Using the 1000 Genomes Dataset

**DOI:** 10.1371/journal.pone.0009366

**Published:** 2010-02-23

**Authors:** Eric R. Gamazon, Wei Zhang, M. Eileen Dolan, Nancy J. Cox

**Affiliations:** 1 Section of Genetic Medicine, Department of Medicine, The University of Chicago, Chicago, Illinois, United States of America; 2 Section of Hematology/Oncology, Department of Medicine, The University of Chicago, Chicago, Illinois, United States of America; 3 Department of Human Genetics, The University of Chicago, Chicago, Illinois, United States of America; Max Planck Institute for Evolutionary Anthropology, Germany

## Abstract

Microarray gene expression data has been used in genome-wide association studies to allow researchers to study gene regulation as well as other complex phenotypes including disease risks and drug response. To reach scientifically sound conclusions from these studies, however, it is necessary to get reliable summarization of gene expression intensities. Among various factors that could affect expression profiling using a microarray platform, single nucleotide polymorphisms (SNPs) in target mRNA may lead to reduced signal intensity measurements and result in spurious results. The recently released 1000 Genomes Project dataset provides an opportunity to evaluate the distribution of both known and novel SNPs in the International HapMap Project lymphoblastoid cell lines (LCLs). We mapped the 1000 Genomes Project genotypic data to the Affymetrix GeneChip Human Exon 1.0ST array (exon array), which had been used in our previous studies and for which gene expression data had been made publicly available. We also evaluated the potential impact of these SNPs on the differentially spliced probesets we had identified previously. Though the 1000 Genomes Project data allowed a comprehensive survey of the SNPs in this particular array, the same approach can certainly be applied to other microarray platforms. Furthermore, we present a detailed catalogue of SNP-containing probesets (exon-level) and transcript clusters (gene-level), which can be considered in evaluating findings using the exon array as well as benefit the design of follow-up experiments and data re-analysis.

## Introduction

Gene expression is an intermediate phenotype that resides between DNA sequence variation and higher-level cellular or whole-body phenotypes including disease susceptibility and individualized drug response. Whole genome expression profiling using high throughput microarray platforms has been a powerful tool used by investigators to create a global picture of cellular function through the quantitative measurement of mRNA of thousands of genes in parallel. Over the past decade, more than 3,000 scientific publications have reported results using the Affymetrix GeneChip arrays (Affymetrix, Inc., Santa Barbara, CA) alone, one of the most frequently used microarray platforms for expression profiling [1].

A typical gene expression profiling study often involves measuring and comparing the relative amount of mRNA expressed in two or more experimental conditions (e.g., normal and diseased, drug treated and untreated). Notably, several recent studies using the International HapMap Project (http://www.hapmap.org/) [2], [3] lymphoblastoid cell lines (LCLs) demonstrated the utility of these microarray platforms in profiling gene expression and dissecting the genetic architecture of gene regulation [4]. For example, using the Illumina BeadChip array (Illumina, Inc., San Diego, CA), Stranger *et al*. [5], [6] profiled and analyzed ∼50,000 transcript targets across the three HapMap populations: CEU (Caucasian residents of European ancestry from Utah, USA), YRI (Yoruba people form Ibadan, Nigeria) and ASN (Han Chinese from Beijing, China and Japanese from Tokyo, Japan). In contrast, Spielman and co-workers [7] used the Affymetrix GeneChip Human Genome Focus array to measure and compare the expression of ∼8,000 genes between the CEU and ASN samples. Taking advantage of the Affymetrix GeneChip Human Exon 1.0ST array (exon array), which was designed to interrogate the entire length of the gene and not just the 3′ end characteristic of conventional oligonucleotide arrays, Zhang *et al*. measured and compared ∼18,000 gene-level transcript clusters [8] and ∼1.4 million exon-level probesets [9] between the CEU and YRI samples. Using these microarray platforms, significant differences in gene expression and alternative splicing between human populations were identified [5]–[9]. Furthermore, common genetic variants, particularly, single nucleotide polymorphisms (SNPs) were found to contribute to a substantial fraction of the natural variation in mRNA amount both within and between human populations [5]–[10]. In addition, genome-wide associations were performed to identify genetic determinants responsible for the cytotoxicities to some anticancer drugs (e.g., etoposide [11], cisplatin [12], carboplatin [13], daunorubicin [14], cytarabine [15]) by integrating the exon array expression data and genotypic data on these HapMap samples [16], [17].

Although appropriate preprocessing approaches such as the RMA (robust multichip average) [18] can be used to adjust the background noise and effects of outliers, identifying expression differences due to hybridization efficiency is critical. The presence of sequence polymorphisms (e.g., SNPs) in target mRNA can cause less efficient hybridization to the microarray probe compared to a perfectly matched reference sequence, potentially leading to reduced signal intensity measurements and resulting in spurious association results [19]. For example, a recent study on the exon array showed that the effect of SNPs was quite severe and could lead to considerable false-positive findings [20]. Though in certain cases, some statistical approaches may be used to detect and account for this effect (e.g., false *cis*-acting expression quantitative trait loci or eQTLs due to polymorphisms in probes [21]), a comprehensive survey of the SNPs in a microarray platform, which is the major aim for this work, may provide a resource to better evaluate the effects of these polymorphisms on microarray expression data.

**Figure 1:**
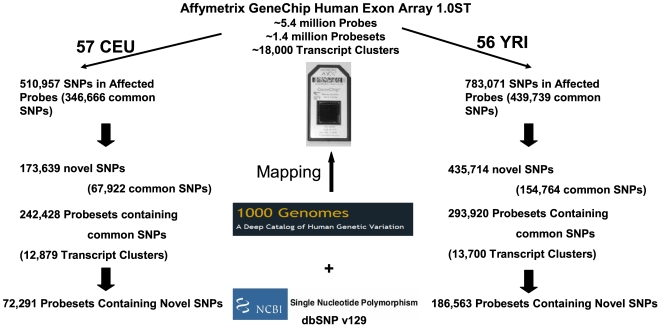
An overview of the methods and major results. The 1000 Genomes Project genotypic data are mapped to the Affymetrix GeneChip Human Exon Array 1.0ST. CEU (Caucasians from Utah, USA) and YRI (Yoruba people from Ibadan, Nigeria) are lymphoblastoid cell lines from the International HapMap Project. Known SNPs are those recorded in the NCBI dbSNP v129 database. Common SNPs are those with minor allele frequencies greater than 5%. Within the exon array, probesets are exon-level and transcript clusters are gene-level.

To date, the publicly available HapMap I/II genotypic data [22] have covered >3.1 million common SNPs. Considering the estimated number of >10 million SNPs in the human genome, it is expected that there may be more unknown/untyped or rarer SNPs in these genotyped HapMap samples. Actually, previous studies have shown that the HapMap genotypic data can not capture a substantial proportion of untyped SNPs [23]–[25], suggesting that deep-resequencing may be needed to uncover more SNPs in the missing regions [26]. Particularly, an unprecedented deep-resequencing project launched in 2008, the 1000 Genomes Project (http://www.1000genomes.org/), ambitiously aims to provide a most detailed map of human genetic variation through genotyping at least 1000 human genomes from world-wide populations using the next-generation sequencing technologies [27], [28]. The specified aims of this project are to identify >95% of the variants with allele frequencies >1% in parts of the human genome that can be sequenced, as well as to identify >95% of the variants with allele frequencies >0.1–0.5% in exons (See the 1000 Genomes Project Meeting Report, http://www.1000genomes.org/bcms/1000_genomes/Documents/1000Genomes-MeetingReport.pdf). In this study, we present a detailed characterization of SNP-containing probesets (exon-level) and transcript clusters (gene-level) of the exon array using the newly released 1000 Genomes Project genotypic dataset (Figure 1). We deposited in the public domain the exon array data on the 176 CEU and YRI HapMap samples from our previous studies of gene expression [8] and transcript isoform variation [9]. We also made available results, using these gene expression data, from our eQTL studies (SCAN database at http://www.scandb.org/) [29] and various pharmacogenomic studies (PACdb at http://www.pacdb.org/) [30]. This new resource of a comprehensive catalogue of SNP-containing probesets and transcript clusters on the exon array can thus help researchers interpret and evaluate findings based on this platform, facilitate future data re-analysis efforts, and benefit the design of follow-up experiments. The same approach can also be applied to other microarray platforms to allow better evaluation of the potential impact of these polymorphisms on microarray expression data.

## Results

An overview of the methods and major results is provided in Figure 1.

### Design of Exon Array

The exon array uses a set of short 25-mer probes to target each feature of interest, together referred to as a probeset. The majority of exon array probesets contain four probes. A gene-level transcript cluster may have one or more exon-level probesets. In total, ∼1.4 million probesets and ∼5.4 million probes under the Affymetrix groupings of “core”, “extended” and “full” were included in our analysis. These probesets cover ∼18,000 “core” transcript clusters, which have RefSeq-supported [31] annotations.

### Mapping SNPs to the Exon Array

Common SNPs were those with MAF (minor allele frequency) greater than 5%. Using the 1000 Genomes Project genotypic data, in total, 510,957 (346,666 with MAF>0.05) SNPs in the CEU samples and 783,071 (439,739 with MAF>0.05) SNPs in the YRI samples (Supplemental Table S1) were found to be located in the >5 million probes of the exon array. Among them, 173,639 (67,922 with MAF>0.05) SNPs in the CEU samples and 435,714 (154,764 with MAF>0.05) SNPs in the YRI samples were novel ones relative to dbSNP v129 (Figure 1). Table 1 shows the categories for those common SNPs with known functional annotations (dbSNP v129) or those that could be imputed based on their neighboring SNPs (Supplemental Table S2 for all SNPs).

**Table 1:** Functional annotations of common SNPs in the exon array.

Function Class^a^	Description	Count (CEU)	Count (YRI)
coding-synonymous	SNPs not causing changes in codons	5009	5110
frameshift	SNPs causing frameshift in coding regions	48	32
intron	SNPs located in introns	117070	122121
missense	SNPs causing changes in codons	4519	4281
near-gene-3	SNPs close to the 3′ of a gene	7547	7721
near-gene-5	SNPs close to the 5′ of a gene	5275	4542
nonsense	SNPs causing STOP codons	53	47
reference	Same as the human genome reference	10189	10182
splice-3	SNPs at the 3′-splice sites	2	3
splice-5	SNPs at the 5′-splice sites	0	1
utr-3	SNPs located in the 3′-untranslated regions	19843	21032
utr-5	SNPs located in the 5′-untranslated regions	3132	2449
NA^b^	Unannotated	173979	262218
**Total**		**346,666**	**439,739**

a: based on dbSNP v129.

b: “NA” includes novel SNPs whose functions have not been classified or imputed.

### SNP-Containing Probesets and Transcript Clusters

**Figure 2:**
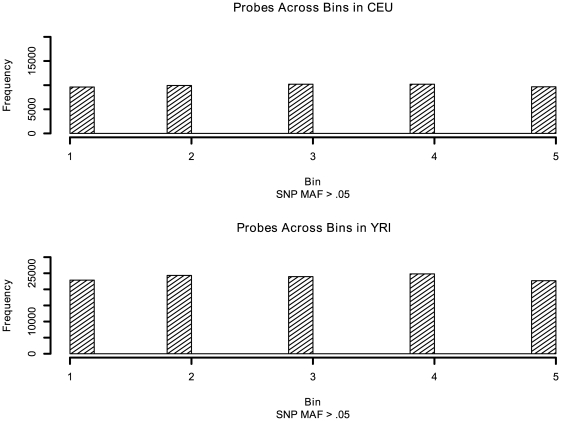
Distribution of SNP locations with the 25-mer exon array probes. Each bin is 5 nt. Left is 5′ and right is 3′. Common SNPs (MAF>0.05) are included. MAF: minor allele frequency.

Focusing on common SNPs (MAF>0.05), among the ∼1.4 million probesets on the exon array, 242,428 probesets (∼17%) (corresponding to 12,879 core-level transcript clusters) in the 57 CEU samples and 293,920 probesets (∼21%) (corresponding to 13,700 core-level transcript clusters) in the 56 YRI samples were found to contain SNPs (Table 2). Supplemental Table S3 lists the statistics for both common and rarer SNPs. In addition, a majority of the SNP-containing probesets harbored only one common SNP (CEU: ∼73%; YRI: ∼69%). There is no chromosomal enrichment in the number of affected probesets in either CEU or YRI samples (binomial test, p<2.2×10^−16^) (Supplemental Figure S1). The complete lists of affected probesets in both populations are provided in supplemental materials (Supplemental Tables S4, S5, S6). In addition, Figure 2 shows the distribution of SNP locations with the exon array 25-mer probes. Affymetrix classifies probesets according to reliability. Since a substantial number of probesets are classified as “extended” and “full”, we determined the number of affected probesets (i.e., containing SNPs with MAF >5%) in each population under the various reliability groupings (Supplemental Table S7). To help evaluate how SNPs in probes may impact gene-level summaries, we provide, in each population, the number of affected probesets (i.e., containing probes with novel SNPs such that MAF >5%) within each transcript cluster (Supplemental Tables S8, S9). To facilitate studies using the array at the probe level, we provide the genomic coordinates of probes containing novel 1000 Genomes SNPs as well as the transcript cluster ID, the probeset ID, each novel SNP's genomic position, and the SNP's position along the probe sequence in each population (Supplemental Tables S10, S11).

**Table 2:** Probesets containing common SNPs based on the 1000 Genomes Project data.

Population	0 SNPs	1 SNP	2 SNPs	≥3 SNPs	Total Affected Probesets
CEU	1183219	177336	40578	24514	242428
YRI	1131727	204650	54179	35091	293920

### Comparison of Affected Probesets with the SNPinProbe 1.0 Database (dbSNP v129)

We previously built a database of SNP-containing probesets in the exon array (SNPinProbes 1.0 [32]) based on dbSNP v129. Approximately 350,000 SNP-containing probesets were filtered out before summarizing gene expression intensities in the CEU and YRI samples (Gene Expression Omnibus Accession: GSE9703 [9], http://www.ncbi.nlm.nih.gov/projects/geo/). To evaluate their potential impact on the previous gene expression data, we compared our new list of SNP-containing probesets and transcript clusters derived from the 1000 Genomes Project with the ∼350,000 affected probesets from the SNPinProbe database (dbSNP v129). The probesets were further grouped based on the number of SNPs in affected probes (e.g., 0, 1, 2, or more SNPs in a particular probeset). Compared with our previous SNPinProbe database [32] based on dbSNP v129, 72,291 and 186,563 probesets in the CEU and YRI samples, respectively, were found to contain novel SNPs from the 1000 Genomes Project (Table 3). In addition, 46,261 probesets in the CEU samples and 101,065 probesets in the YRI samples were found to contain both known and novel SNPs. The proportions of new affected probesets relative to the total ∼1.4 million probesets are, therefore, ∼8% for the CEU samples and ∼20% for the YRI samples.

**Table 3:** Comparison with the SNPinProbe database(dbSNP v129).

Population	Class	Novel	Known	Both	Total
**CEU**	1 SNP	59462	159789	0	219251
	2 SNPs	8677	31472	19303	59452
	≥3 SNPs	4152	11706	26958	42816
	Total	72291	202967	46261	321519
**YRI**	1 SNP	142719	125199	0	267918
	2 SNPs	30529	22148	40422	93099
	≥3 SNPs	13315	6509	60643	80467
	Total	186563	153856	101065	441484

Both: affected by novel and known SNPs.

### Potential Influence of SNPs on Differentially Spliced Probesets

**Figure 3:**
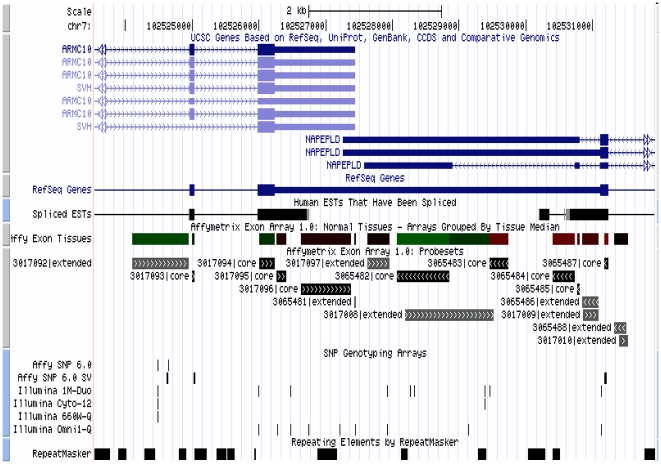
A differentially spliced probeset that could be affected by novel SNPs. The probeset 3017096 (chr7: 102526627–102527377) has a novel SNP (chr7: 102526628) in the CEU samples. The probeset was originally found to be differentially spliced between the CEU and YRI samples. UCSC Genome Browser (http://genome.ucsc.edu/) was used to plot the genomic positions (hg18, March, 2006) of the probeset in relation to the other probesets for the *ARMC10* gene. This novel SNP lies within the probe chr7:102526627–102526651.

Among the 782 differentially spliced probesets from a previous study [9], 24 probesets were found to contain at least one novel SNP in the CEU samples, and 94 probesets were found to contain at least one novel SNP in the YRI samples. This analysis includes both common (MAF >0 .05) and rare novel SNPs in affected probesets, thus yielding the worst-case scenario. Therefore, up to 15% of the 782 identified probesets could be affected by the novel SNPs from the 1000 Genomes Project. Supplemental Table S12 lists these potentially affected probesets, the number of novel SNPs, and the number of rare SNPs (MAF <0.05) among the novel SNPs, in each population. A majority (∼72%) of these probesets contained one novel SNP. Therefore, among the affected differentially spliced probesets, most would still yield expression estimates after filtering for SNP-containing probes. Potential lost coverage in these probesets is minimal. Figure 3 illustrates an example.

## Discussion

Various factors (e.g., sample preparation, reagent quality) may influence hybridization of target mRNA to microarray probes, thus causing unreliable measurements of expression intensities. Many of these technical factors can be optimized or controlled. However, due to the existence of genetic variation among individuals, polymorphisms in some samples may cause less efficient hybridization to microarray probes, which are often designed based on the reference sequences. Though a couple of recent studies considered the effect of polymorphisms in probes before summarizing expression data [8], [9], [33], it has been difficult to comprehensively investigate the potential impact of common SNPs on microarray expression platforms, partly because of the lack of a detailed map of human genetic variation. The newly released genotypic data from the 1000 Genomes Project provides an opportunity to systematically evaluate the potential influence of common genetic variants on these microarray platforms for their use in human samples. Our aim, therefore, was to utilize the 1000 Genomes Project genotypic data to evaluate the distribution of SNPs, especially those common SNPs (MAF>0.05) on the Affymetrix exon array, which has recently been used in gene expression [8], transcript isoform variation [9], [33] as well as numerous pharmacogenomic studies [16], [17]. Because of the emerging stage of the next-generation sequencing technologies, systematic biases and data variability may need to be considered when utilizing these data [34]. Focusing on common SNPs, therefore, could potentially alleviate the problem of errors in base calling.

Focusing on common SNPs with MAF>0.05, overall, ∼17% and ∼21% probesets out of the total ∼1.4 million probesets were found to contain at least one common SNP in their probes for the CEU and YRI samples, respectively (Table 2). The YRI samples had many more SNP-containing probesets than the CEU samples, consistent with the hypothesis that Africans are an older population with more genetic variation [35]. It has been observed that probe-level expression can have significant changes when a polymorphism is present near the middle of the target area (i.e., between positions 6 and 21 of a 25-mer probe) [20]. The distribution of the SNPs–both novel (with MAF>0.05) as well as novel in general–in the exon array probes appeared to be evenly distributed across the probe length (Figure 2), suggesting that roughly 60% (corresponding to bins 2–4 in Figure 2) of the SNP-containing probes could be affected more significantly by the presence of SNPs. A majority of those known SNPs (based on dbSNP v129) and the novel SNPs, whose positions allowed reliable functional imputation, are located in the intronic regions (CEU: ∼68%; YRI: ∼69%) (Table 1).Those intronic SNP-containing probes may, therefore, particularly affect the measurement of expression of a novel exon not present in the reference sequence. Similarly, for example, the SNPs located in UTRs may affect the detection of alternative initiation or termination.

Conceptually, the 1000 Genomes Project genotypic data aims to cover both known SNPs and novel SNPs. Our next aim was to evaluate how the 1000 Genomes Project genotypic data was compared with the known dbSNP data. Particularly, we previously built the SNPinProbe database [32] of all affected exon array probesets based on the then current dbSNP v129. Both gene-level [8] and exon-level [9] expression data taking into account these SNP-containing probesets have been made public. Therefore, a comparison between what we found with the 1000 Genomes Project genotypic data and the SNPinProbe database [32] would allow us to evaluate the potential influence of novel SNPs on our previous gene expression datasets [8], [9] and the results based on those expression data (e.g., pharmacogenomic discoveries [16], [17]). The SNPinProbe database is comprised of ∼350,000 affected probesets (combined CEU and YRI samples) [32]. In contrast, using the 1000 Genomes Project data, ∼320,000 (∼242,000 with MAF>0.05) and ∼440,000 (∼294,000 with MAF>0.05) affected probesets were identified for the CEU and YRI samples, respectively. If the two populations were combined as the SNPinProbe database did, 506,872 probesets would be identified to contain at least one SNP. Therefore, ∼157,000 more probesets would be identified using the 1000 Genomes Project data, suggesting that interpretation of any association results including these probesets should take into account the potential confounding effect of polymorphisms.

To further illustrate the potential effect of any probesets affected by novel SNPs as well as to demonstrate an application of this new resource of SNP-containing exon array probesets, we examined a list of 782 differentially spliced probesets between the CEU and YRI samples [9]. Among the 782 identified probesets, ∼15% would be found to contain at least one novel SNP in one population. For example, the probeset 3017096 (chr7: 102526627–102527377), which interrogates *ARMC10* together with probesets 3017094, 3017095, and 3017088, has a novel SNP (chr7: 102526628) in the CEU samples (Figure 3). The identification of its alternative splicing between the two populations, therefore, should be cautiously evaluated. For this particular study (alternative splicing between human populations [9]) though, it appeared that most of the identified differentially spliced probesets (∼85%) would be free of any known or novel SNPs. Of the remaining probesets (∼15%), a majority (∼72%) would nevertheless allow expression estimates derived from probes unaffected by SNPs. In addition, *ARMC10* showed evidence of being differentially spliced between CEU and YRI (p = 0.015, two-tailed *t*-test) when the probeset 3017096 is included, but shows no evidence (p = 0.48, two-tailed t-test) when the SNP-containing probes are excluded.

In summary, due to the amount of targets on a single microarray, obviously, a systematic experimental evaluation of the effects of polymorphisms on gene expression would be extremely difficult. Though statistical approaches may be used to adjust these effects in certain cases [21], a comprehensive catalogue of the SNP affecting microarray probes, however, can allow researchers to immediately evaluate previous findings based on these gene expression data, facilitate future data re-analysis efforts (e.g., removing affected data points), and benefit the design of follow-up experiments (e.g., to prioritize candidates by avoiding those potentially affected genes). In addition, our analysis showed an application of the newly released 1000 Genomes Project genotypic data which clearly will benefit the entire community of biomedical research (e.g., pharmacogenomics [36]) by providing a detailed map of human genetic variation. Finally, similar analyses could be performed on other microarray platforms in the future to allow a cross-platform comparison.

## Materials and Methods

### Affymetrix Exon Array Annotations

The probe, probeset (exon-level) and transcript cluster (gene-level) annotations for the Affymetrix GeneChip Human Exon 1.0ST array were downloaded from the Affymetrix NetAffx Analysis Center (http://www.affymetrix.com/analysis/index.affx) (NetAffy build 27). The human genome reference version is NCBI build 36 (hg18, March, 2006).

### The 1000 Genomes Project Data

The 1000 Genomes Project was launched in January, 2008. The first set of SNP calls of four genomes of HapMap LCLs (3 samples from a CEU parents-child trio and 1 YRI sample) were released Dec., 2008 from the high coverage (>20×) pilot. The SNP calls on the CEU trio (father: NA12891; mother: NA12892; child: NA12878) were based on the Illumina platform (mostly paired end 35 bp reads). The SNP calls on the YRI sample (NA19240) were detected using the Applied Biosystems SOLiD (Sequencing by Oligo Ligation and Detection) sequencing platform. More recently, sequence data and SNP calls on >600 genomes (April, 2009) in the low coverage pilot were also released. We downloaded the 1000 Genomes Project data of the currently available 57 CEU samples and 56 YRI samples including FASTQ files (nucleotides and quality assessments), SNP calls, and Binary Simple Alignment/Map files (BAM) (ftp://ftp-trace.ncbi.nih.gov/1000genomes), as well as FASTA files for the human genome reference assembly (ftp://ftp.ensembl.org/pub/current_fasta/homo_sapiens/dna/). We wrote our own Extractor (for the FASTQ files) and Analyzer (for summarization), and invoke the tool (SAMtools, http://samtools.sourceforge.net/samtools-c.shtml) for the (binary) Sequence Alignment/Map format (for multiple alignments) used by the Sanger Institute. Summary analysis data are stored in a relational data store (MySQL, http://www.mysql.com).

### Mapping 1000 Genomes SNPs to the Exon Array

The genomic regions of the probesets, along with transcript cluster annotations, were loaded into MySQL to be easily queried. The genomic positions of >9 million SNPs in the 57 CEU samples and of >13 million SNPs in the 56 YRI samples in the 1000 Genomes data release were mapped to dbSNP v129 and RefSeq genes based on the reference assembly (build 36). This facilitated the annotation of known SNPs with rs identifier (dbSNP v129), RefSeq [31] alleles, functional class, and host gene, as well as enabled the identification of novel SNPs. We imputed the host gene for intragenic SNPs based on the genomic coordinates found in RefSeq [31] Gene, as well as the functional class for novel SNPs in the following cases: (a) a SNP with flanking 5′-UTR (untranslated region) neighbors annotated to the same gene was assigned the same functional designation; similarly, for 3′-UTR (b) a SNP with flanking “near-gene-5′” or “near-gene-3′” neighbors annotated to the same gene (within 2000 bases) received the same functional designation. Genome-wide queries were then performed between the >17 million SNPs and the ∼1.4 million probesets.

### Potential Influence on Differentially Spliced Probesets

We previously identified a list of 782 differentially spliced probesets between the CEU and YRI samples [9] by filtering out ∼ 600,000 SNP-containing probes in ∼350,000 probesets as in the SNPinProbe database [32]. The exon array data is MIAME compliant and that the raw data has been deposited in the NCBI Gene Expression Omnibus (GSE9703, http://www.ncbi.nlm.nih.gov/geo/). To evaluate the potential influence of any novel SNPs on our previous results, we compared the 782 differentially spliced probesets with our newly identified SNP-containing probesets. Particularly, probesets affected by novel SNPs in only one population (either CEU or YRI) were compared with the 782 probesets, assuming a comparison between the CEU and YRI samples could be especially biased due to the existence of SNPs in one population.

## Supporting Information

Figure S1 Chromosomal distribution of affected core-level probesets in the exon array. Blue indicates the CEU samples; Red indicates the YRI samples. Common SNPs (MAF>0.05) are included. MAF: minor allele frequency.
(1.41 MB EPS)

Table S1 Chromosomal distribution of all SNPs in the exon array.
(0.02 MB XLS)

Table S2 Functional annotations of all SNPs in the exon array.
(0.02 MB XLS)

Table S3 SNP-containing probesets based on the 1000 Genomes Project genotypic data.
(0.01 MB XLS)

Table S4 A list of SNP-containing probesets (exon-level) for the CEU samples.
(1.30 MB TXT)

Table S5 A list of SNP-containing probesets (exon-level) for the YRI samples.
(1.02 MB TXT)

Table S6 Chromosomal distribution of probesets with common SNPs.
(0.01 MB XLS)

Table S7 The breakdown of affected probesets under the Affymetrix reliability groupings (“core”, “full”, “extended”).
(0.01 MB XLS)

Table S8 The number of probesets containing probes with novel and common SNPs within each transcript cluster (CEU).
(0.49 MB TXT)

Table S9 The number of probesets containing probes with novel and common SNPs within each transcript cluster (YRI).
(1.32 MB TXT)

Table S10 Probe-level information of probesets containing novel SNPs (CEU).
(2.46 MB TXT)

Table S11 Probe-level information of probesets containing novel SNPs (YRI).
(2.42 MB TXT)

Table S12 The differentially spliced probesets that could be affected by novel SNPs.
(0.00 MB TXT)
